# Cortical Cross-Frequency Coupling Is Affected by *in utero* Exposure to Antidepressant Medication

**DOI:** 10.3389/fnins.2022.803708

**Published:** 2022-03-03

**Authors:** Anton Tokariev, Victoria C. Oberlander, Mari Videman, Sampsa Vanhatalo

**Affiliations:** ^1^Department of Clinical Neurophysiology, BABA Center, New Children’s Hospital, Helsinki University Hospital, University of Helsinki, Helsinki, Finland; ^2^Neuroscience Center, Helsinki Institute of Life Science, University of Helsinki, Helsinki, Finland; ^3^Department of Computer Science, Aalto University, Espoo, Finland; ^4^Department of Pediatric Neurology, New Children’s Hospital, Helsinki University Hospital, University of Helsinki, Helsinki, Finland; ^5^Department of Physiology, University of Helsinki, Helsinki, Finland

**Keywords:** infant, EEG, brain network, antidepressant, SRI, neurodevelopment, depression, pregnancy

## Abstract

Up to five percent of human infants are exposed to maternal antidepressant medication by serotonin reuptake inhibitors (SRI) during pregnancy, yet the SRI effects on infants’ early neurodevelopment are not fully understood. Here, we studied how maternal SRI medication affects cortical frequency-specific and cross-frequency interactions estimated, respectively, by phase-phase correlations (PPC) and phase-amplitude coupling (PAC) in electroencephalographic (EEG) recordings. We examined the cortical activity in infants after fetal exposure to SRIs relative to a control group of infants without medical history of any kind. Our findings show that the sleep-related dynamics of PPC networks are selectively affected by *in utero* SRI exposure, however, those alterations do not correlate to later neurocognitive development as tested by neuropsychological evaluation at two years of age. In turn, phase-amplitude coupling was found to be suppressed in SRI infants across multiple distributed cortical regions and these effects were linked to their neurocognitive outcomes. Our results are compatible with the overall notion that *in utero* drug exposures may cause subtle, yet measurable changes in the brain structure and function. Our present findings are based on the measures of local and inter-areal neuronal interactions in the cortex which can be readily used across species, as well as between different scales of inspection: from the whole animals to *in vitro* preparations. Therefore, this work opens a framework to explore the cellular and molecular mechanisms underlying neurodevelopmental SRI effects at all translational levels.

## Introduction

Up to 5–8% of newborn infants are exposed to serotonin reuptake inhibitors (SRIs) used for treating major depressive disorder (MDD), anxiety disorders, and eating disorders, all of which commonly require medical care during pregnancy (Andrade et al., 2008; Hanley and Mintzes, 2014; Charlton et al., 2015; Dawson et al., 2015). Such widespread use of SRIs during pregnancy is justified by the concerns that an untreated antenatal depression would affect early development of the offspring (Hanley et al., 2015; Gentile, 2017; Kautzky et al., 2021) while the SRIs are not known to associate with major teratogenic effects other than possible cardiac issues (Reefhuis et al., 2015; Wemakor et al., 2015; Wisner et al., 2020; Kolding et al., 2021). However, recent clinical findings have challenged the current liberal practice showing that newborn infants exposed to SRIs *in utero* show markedly high rates of SRI withdrawal symptoms requiring medical attention (Ulbrich et al., 2021; Wang and Cosci, 2021). In addition, recent studies on magnetic resonance (MR) imaging (Lugo-Candelas et al., 2018; Rotem-Kohavi et al., 2019) and cortical activity (Videman et al., 2017) have suggested that SRIs may clearly have measurable effects, prompting further studies on the associated brain mechanisms.

The rapidly accumulating data from experimental neuroscience shows that the SRI target, serotonin transporter, serves crucial ontogenetic roles during fetal brain development (Nordquist and Oreland, 2010; Bourke et al., 2013), including guidance of neuronal migration and growth of neuronal networks (Borue et al., 2007; Homberg et al., 2010; Kiryanova et al., 2013). Rodent pups exposed to SRI *in utero* exhibit distorted cortical and subcortical microstructures and function (Persico et al., 2001; Xu et al., 2004; Liao and Lee, 2011; Simpson et al., 2011; Suri et al., 2015). Since a direct long-leap translation from cellular-level experimental findings to pharmacological treatments of human infants is challenging (Durrmeyer et al., 2010), it has become apparent that better and more specific translational bridges, or biomarkers, are needed from experimental findings to human infants.

The potential translational biomarkers should be based on neurobiological mechanisms derived from measures of brain structure or neuronal function. Structural studies with diffusion tensor MR imaging have become popular in assessing pathologies in human subjects, including newborn infants (Pecheva et al., 2018). However, the currently available animal literature suggests that cellular level effects of SRIs are found in the intracortical microstructure (Persico et al., 2001; Xu et al., 2004; Liao and Lee, 2011; Simpson et al., 2011; Suri et al., 2015), which is an order of magnitude smaller detail than what can be genuinely studied with MR imaging. In addition, the cellular level correlates of the MR results are too poorly known to allow their use as a genuinely translational biomarker (Jones et al., 2013; Sarwar et al., 2021). Instead, newborn brain function can be studied directly by measuring neuronal population activity with scalp electroencephalography (EEG) or indirectly by measuring blood-oxygen-level-dependent (BOLD) signal fluctuations with an fMRI method. In the newborn infant, EEG only provides an estimate of the fluctuations in spontaneous neuronal activity (Kozberg et al., 2016). Hence, EEG is the only viable option for measuring SRI effects in a way that can be directly translated between human infants and experimental animal models, both *in vivo* and *in vitro*.

Next, there is a need to identify metrics of EEG that allow translation across many experimental levels and recording settings. Prior cortical recordings in animal models with extracellular electrodes have shown SRI effects on the spatial activity correlations in cortical neuronal ensembles (Liao and Lee, 2011; Simpson et al., 2011; Zhou et al., 2015), while prior clinical research on human infant EEG after SRI exposure showed changes in signal power, synchrony and phase-amplitude coupling (Videman et al., 2017). While these electrophysiological results between species are compatible with each other, the examined neuronal mechanisms are different, and the levels of inspection differ by orders of magnitude in terms of spatial scales. Nevertheless, the existing literature suggests some characteristics for the measures that could provide translation between species and across multiple levels of inspection. First, the measures should be able to directly assess cortical neural activity from the scalp EEG recordings of the infants. This would facilitate the comparison of findings between non-invasive human recordings and invasive recordings in animal models. Second, the measures should examine functional interactions within local and large-scale neuronal networks. Third, the measures should estimate the interplay between neural oscillations at different frequencies, which underpins the intrinsic mechanisms guiding the early brain networking (Vanhatalo et al., 2005; Tokariev et al., 2016b; Mariscal et al., 2021). All these requirements can be met by studying cortical activity—estimated from reconstruction of the scalp-recorded EEG—and analysis of phase-phase correlations (PPC) for remote cortico-cortical interactions, and phase-amplitude coupling (PAC) for local interactions. PPC is considered to support large-scale communications at high temporal precision and is sensitive to various adversities including drug exposure (Palva et al., 2005, 2010; Engel et al., 2013; Tokariev et al., 2019b,^2021^). PAC is taken as a measure of cross-frequency interaction that is found in most brain functions and structures (Cohen et al., 2009; Canolty and Knight, 2010; Liu et al., 2015; Palva and Palva, 2018; Siebenhühner et al., 2020). Due to its spatially integrating role within local cortical networks, it is particularly strong in the early developing brain networks in both human EEG (Vanhatalo et al., 2005; de Camp et al., 2017; Moghimi et al., 2020; Shibata and Otsubo, 2020; a.k.a. nested oscillations) and various animal models (Minlebaev et al., 2007; Colonnese et al., 2010; Murata and Colonnese, 2016; Li et al., 2017; Molnár et al., 2020), including a spontaneous emergence in even developing brain organoids (Trujillo et al., 2019). Hence, PAC could offer an ideal, widely translatable, and mechanism-based generic measure of brain function. Taken together, PPC and PAC are two different neuronal interaction mechanisms that coordinate neural activity along spatial and laminar dimensions, therefore, presumably playing an important role in the early activity-driven development of brain networks.

Here, we hypothesized that *in utero* exposure to SRIs in the human newborn infants would change cortical frequency-specific and cross-frequency interactions measured by PPC and PAC, respectively. This hypothesis was motivated by the global-level PAC findings from the scalp EEG data in the same infant cohort (Videman et al., 2017), as well as by the many other PPC findings in infants after other drug exposures or neonatal adversities (Tokariev et al., 2019b,^2021^; Yrjola et al., 2021). All these prior studies have suggested that PAC and PPP may be sensitive in disclosing early neurodevelopmental effects. Moreover, we hypothesized that these effects may also have a link to later neurocognitive development of the exposed infants. To test this hypothesis, we designed a novel pipeline for spatially resolved PPC and PAC assessments of the newborn cortical activity, and we re-examined a previously published clinical EEG dataset (Videman et al., 2017) from a cohort of infants exposed to SRIs *in utero* with a long-term neurodevelopmental follow-up.

## Materials and Methods

### Study Design Overview

We used a previously collected EEG data (Videman et al., 2017) during active sleep (AS) and quiet sleep (QS) from two groups: healthy controls (HC) and infants those were *in utero* exposed to antidepressants (SRI). Electroencephalographic signals were source reconstructed and band-pass filtered into 24 frequency bands of interest. Next, we computed frequency-specific PPC networks and analyzed their interactions when transitioning between sleep states in both groups. We also estimated PAC at two scales: whole-brain and source-level across multiple combinations of frequency bands (nesting vs. nested). Connectivity changes of PPC interaction patterns and PAC strength within group contrasts were further correlated to later cognitive outcomes of SRI subjects.

### Subjects and Data Collection

This cohort was initially collected for a study that examined the effects of *in utero* SRI exposure on the early development of cortical activity and infant’s early neurodevelopment (Videman et al., 2017). Scalp EEG data were collected from two groups of infants during day sleep in the Helsinki University Central Hospital (Finland). One group included infants exposed to SRI *in utero* (SRI, N_SRI_ = 22) and other group comprised healthy controls without any known medical incidents (HC, N_HC_ = 67). Subjects in both groups were born full-term at gestational age of 39.9 ± 1.1 weeks and 40.3 ± 1.1 weeks (mean ± standard deviation, SD) respectively, with no significant group difference. More detailed clinical information about the subjects can be found in Videman et al. (2017).

The EEG recordings were performed mainly using NicOne EEG amplifier (Cardinal Healthcare/Natus, United States) and Waveguard caps (sintered Ag/AgCl electrodes; ANT-Neuro, Germany) with 19–28 sensors which were placed according to the International 10–20 standard. To enable sleep state classification (into active and quiet sleep) we also included chin electromyogram, electrocardiogram, eye movements and respiratory data (André et al., 2010).

Cognitive development of SRI babies was evaluated at the age of two years (24.3 ± 0.4 months) by an experienced psychologist using neurodevelopmental assessment according to Bayley Scales of Infant and Toddler Development (BSID-III; Bayley, 2006). For a representative measure of neurodevelopmental outcome, we chose to use the standardized scores of the Cognition domain in our correlation analysis with the EEG metrics NC and PPC.

The study was approved by the Ethics Committee of the Helsinki University Central Hospital. Also, the informed consent for each individual case was received from a parent or guardian before the data collection.

### Serotonin Reuptake Inhibitor Medication

As described in our previous work (Videman et al., 2017), the detailed time courses of the *in utero* SRI exposures were documented from the patient reports, and for ethical reasons, they were not modified for the study purposes. The average daily doses of administered medications were as follows: citalopram (six mothers, 17.5 mg), sertraline (six mothers, 47.5 mg), escitalopram (six mothers, 8 mg), venlafaxine (two mothers, 75 mg), paroxetine (two mothers, 30 mg), duloxetine (one mother, 60 mg), and mirtazapine (one mother, 15 mg). Out of 22 mothers, four reported being on SRI polytherapy, 16 used the medication throughout pregnancy, while 21 got the medication during the first and the second trimester only. One mother finished and one started medication in the end of the second trimester, while four finished it during the last two to four weeks prior to delivery.

### Electroencephalogram Pre-processing

First, sleep EEG data were classified into periods of AS and QS according to the conventional criteria (André et al., 2010). We also selected the same 19 channels (Fp1, Fp2, F7, F3, Fz, F4, F8, T7, C3, Cz, C4, T8, P7, P3, Pz, P4, P8, O1, and O2) for all subjects to enable group comparison. Next, we accumulated 3-min-long epochs of artifact-free EEG from each sleep state for each subject. In further analysis we included only subjects that had EEG data of sufficient length and quality at both states. This led to the final samples of N_SRI_ = 19 infants in SRI cohort and N_HC_ = 61 infants in HC cohort. At the time of EEG, the conceptional ages of the newborns in these groups were 42.3 ± 0.8 weeks and 42.2 ± 0.9 weeks, respectively, with no significant difference (*p* = 0.75, Wilcoxon rank-sum test). Further, EEG epochs were band-pass filtered within 0.4–45 Hz, down-sampled into the same sampling frequency Fs = 100 Hz (from initial 250 and 500 Hz) and converted to average montage. Finally, pre-processed EEG signals were filtered into 24 frequency bands covering the range 0.4–38 Hz. The first central frequency (Fc) was set to 0.5 Hz and all consecutive central frequencies were computed as 1.2⋅Fc relative to previous one. Cut-offs for all frequency bands were taken as 0.85⋅Fc and 1.15⋅Fc, whereas stop-band frequencies were set to 0.5⋅Fc and 1.5⋅Fc. Such approach allows generating 50% overlapping frequency bands those of quasi equal width on a logarithmic scale (Tokariev et al., 2019a). All band-pass filtering was done off-line using pairs of the corresponding low- and high-pass Butterworth filters and in forward-backward directions.

### Computation of Cortical Signals

**FIGURE 1:**
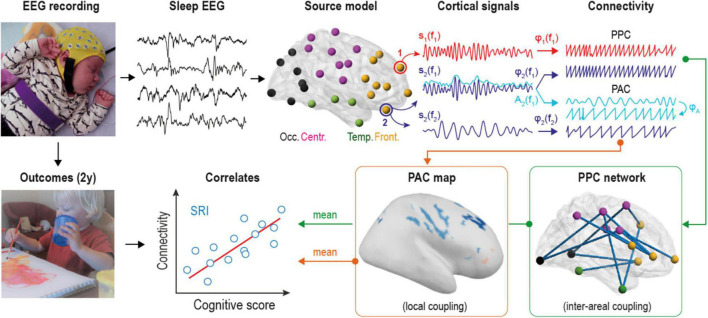
Overview of the analytical pipeline. Multi-channel electroencephalographic (EEG) data was collected during daytime sleep (at both active and quiet states) from two groups of infants. Scalp EEG was converted into 58 cortical source signals and filtered into 24 frequency bands to estimate both phase-phase correlations (PPC) and phase-amplitude coupling (PAC) connectivity modes at each frequency. PPC measures coupling between different cortical sources, while PAC estimates cross-frequency coupling within the given source. Mean connectivity from statistically significant group contrasts was correlated to neurocognitive outcomes of serotonin reuptake inhibitors (SRI) -exposed infants at two years of age.

Electroencephalographic (EEG) signals were converted into cortical signals using realistic infant head model (Tokariev et al., 2016a,2019b). The model included three outer surfaces (scalp, skull, intracranial volume; 2562 vertices per each), cortex as the source space (with 8014 orthogonal dipoles), and 19 EEG sensors located according to the recording setup. Important, that the model accounted realistic infant tissue conductivities: 0.43 S/m for scalp, 0.2 S/m for skull and 1.79 S/m for intracranial fluid (Despotovic et al., 2013; Odabaee et al., 2014). The forward operator was computed using symmetric boundary element method (Gramfort et al., 2010), whereas the inverse operator was obtained with dynamic statistical parametric mapping (Dale et al., 2000) as it is implemented in Brainstorm (Tadel et al., 2011). All individual cortical sources were clustered into 58 brain regions according to special infant parcellation^1^ scheme (Tokariev et al., 2019b; see also Figure 1). Parcels were also grouped into four anatomical categories: frontal, central, temporal, and occipital. Notably, the accuracy of whole-brain source reconstruction depends on the number of recording electrodes (Tokariev et al., 2016a), so the source model computed from the standard clinical montage with 19 sensors is not able to fully represent all sources in our cortical surface model. This limitation needs to be carefully taken into account in the subsequent analysis, hence we employed a previously developed simulation-based procedure that evaluates the fidelity of individual sources in the specific head model and assign them corresponding coefficients (Tokariev et al., 2019b). The signals from each cortical parcel were computed as the mean of all source signals within them weighted by these fidelity coefficients. Such approach allows suppressing the contribution of non-reliable sources and relying mostly on the information from good quality sources.

### Analysis of Phase-Phase Coupling Networks

**FIGURE 2:**
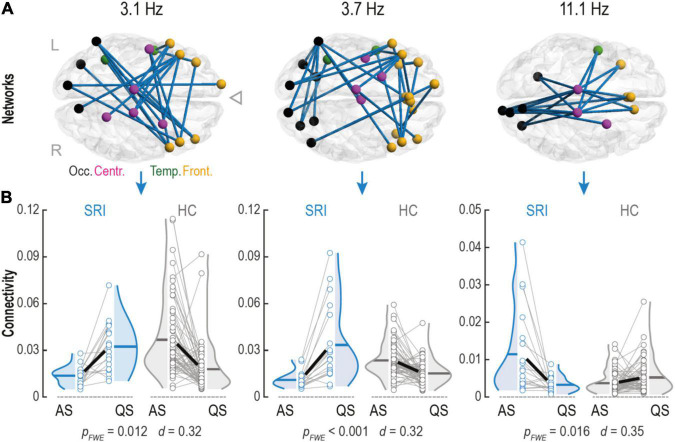
Serotonin reuptake inhibitor (SRI) exposure impacts connectivity changes within phase-phase correlation (PPC) networks in infants during sleep. **(A)** Cortical PPC networks of infants that were *in utero* exposed to SRI showed significant sleep-by-group interactions at theta (Fc = 3.1 Hz and Fc = 3.7 Hz) and alpha (Fc = 11.1 Hz) frequencies. Notably, that theta networks predominantly connected frontal cortices which were also making long-range projections to occipital lobe. Alpha network connected central areas to frontal and occipital cortices and was constrained mostly to midline. Cortical regions on the glass brains are marked with different colors: occipital (black), temporal (green), central (purple), and frontal (orange). **(B)** Sleep dynamics in SRI group was reversed during transitions between two sleep states (AS vs. QS) compared to healthy controls (HC): in theta networks connectivity increased during quiet sleep (QS) (p_FWE_ ≤ 0.012 for both), whereas in alpha network connectivity decreased in QS (p_FWE_ = 0.016). Interactions were tested with paired two-tailed *t*-test followed by permutation-based correction for family wise error (FWE) rate. Effect size was estimated as the mean of Cohen’s d values across all significant connections.

We computed pairwise PPC between all cortical areas using debiased weighted phase lag index (Vinck et al., 2011) which is insensitive to volume conduction (Palva and Palva, 2012; Palva et al., 2018). This resulted in a set of individual frequency- and sleep state-specific PPC networks, where cortical parcels were considered as nodes, and PPC strength was taken as functional connections (or edges). Next, we corrected each network by removing edges that cannot be reliably estimated from the recording setup with 19 EEG sensors. For this purpose, we employed a simulation-based procedure that contrasts each individual edge from the synthetic networks to their copies that were reconstructed with the particular head model (see Tokariev et al., 2019b for details). The procedure outputs a binary template that rejects the same subset of unreliable connections (about 32%) from all empirical networks. Then, in line with our previous work (Tokariev et al., 2019a,^2021^), we focused on the sleep-by-group interaction that holds important information about infant brain function (Figure 2). To isolate frequency-specific network patterns with altered sleep-related dynamics due to SRI exposure, we used network-based statistics (Zalesky et al., 2010). The t-statistics (threshold 2.5) was applied to individual edges and followed by permutation-based family wise error (FWE) rate correction procedure for the significant network components (alpha level 0.05, 5000 permutations).

### Analysis of Phase-Amplitude Coupling

#### Global Phase-Amplitude Coupling

Band-filtered parcel signals were split into two sets: “nesting” low-frequency components (Fc = 0.5 Hz, …, 2.1 Hz) and “nested” high-frequency components (Fc = 3.1 Hz, …, 33.1 Hz). Next, using Hilbert transform, we computed instantaneous phase of the nesting component and amplitude envelope of the nested component. Further, amplitude envelope of the nested component was band-pass filtered with the same filter as the nesting component of interest and the phase was extracted also via Hilbert transform (see also Figure 1). The phase synchrony between nesting component and the filtered amplitudes of nested component was estimated using phase locking value (Lachaux et al., 1999); what resulted is a metric known as nestedness coefficient (NC),(Vanhatalo et al., 2004; Tokariev et al., 2016b). We computed NC values for all cortical parcels (N_p_ = 58) and then took their mean to get global measure representing whole-brain PAC mode for each combination of nested-vs-nesting frequencies (overall 126 combinations). Individual frequency-frequency PAC maps were also averaged across each group during distinct sleep states separately. We statistically compared global NC values between groups (SRI vs. HC) using Wilcoxon rank sum test and between sleep states (AS vs. QS) using Wilcoxon signed-rank test (alpha level 0.05 for both cases). The multiple comparisons in each case were controlled with Benjamini-Hochberg procedure.

#### Source-Level Phase-Amplitude Coupling

**FIGURE 3:**
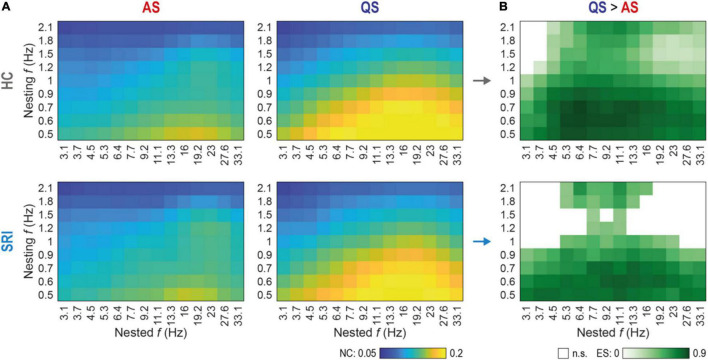
Sleep-related changes in global phase-amplitude coupling (PAC) are less discriminative in SRI infants. **(A)** Spectral fingerprints of global PAC for healthy controls (HC, top row) and exposed to serotonin reuptake inhibitors (SRI, bottom row) infants at active sleep (AS, left column) and quiet sleep (QS, right column). Colors show the group mean nestedness coefficient (NC) computed for the whole cortex as a function of nested and nesting frequencies. There were no significant differences in state-specific PAC values between HC and SRI (Wilcoxon rank sum test). The peak in AS was more focal and mostly constrained to interactions of lower nesting frequencies vs. mid-range nested frequencies. During QS peak reflects to more broadband interaction with stronger coupling. **(B)** Discrimination between sleep states as a function of SRI exposure. Shades of green code the effect size (ES; estimated with rank-biserial correlation) of significant differences (QS > AS) in global PAC between sleep states within each group (*p* < 0.03 for all tests, Wilcoxon signed-rank test, FDR corrected). White cells mark non-significant (n.s.) cases. Notably, that SRI group shows suppressed discrimination at higher nesting frequencies, compared to broadband effect in HC.

To test if there were any spatially constrained differences between groups that were not seen on the global level, we estimated PAC at the level of cortical sources (N_s_ = 8014). The procedure was similar to parcel-level NC computation, but the individual source signals were used. The spectral distribution of NC values (Figure 3) motivated us to collapse multiple nesting frequencies into single broader range 0.4–1.4 Hz. To reduce computational load, we used only Fc = 5.3, 7.7, 11.1, 16, and 23 Hz as nested frequencies. From this analysis we excluded sources that had fidelity weights lower than median value of the fidelity operator (“technical correction”). This procedure rejected sources that are in the “blind spot” for 19-channel EEG cap and cannot be estimated reliably (about 50%). The rest of the sources were compared between two groups in their NC levels with two one-tailed Wilcoxon rank sum tests (SRI > HC and SRI < HC, alpha level 0.05). Sources that showed significant difference were clustered into spatial components. Isolated significant sources, that had no neighbors along the cortical surface, were also excluded from the further analysis. To control for FWE rate, we adapted the idea from the cluster-based correction approach (Maris and Oostenveld, 2007) to source-level NC maps. Mean effect size (estimated with rank-biserial correlation) of each NC component observed in empirical data was compared to the distribution of the effect sizes obtained after 5000 permutation tests for the same component. Components below the 95th percentile were considered as non-significant.

### Relation to Neurodevelopment

To test if the group differences relate to later neurodevelopment, we correlated (Pearson test) connectivity strength from the groups’ contrasts to Bayley cognitive scores in SRI infants (N_SRI_ = 15). For PPC networks we used sleep-related connectivity changes (mean strength at AS minus mean strength at QS) within interaction networks. For PAC we averaged nestedness coefficients across all significantly different areas. We used Benjamini-Hochberg procedure for false discovery rate (FDR) correction across all connectivity-to-outcome tests.

### Methodological Considerations

Our study was prospective with a stringent psychiatric evaluation of the maternal state (Videman et al., 2017), which provides a good clinical characterization of the patient cohort. Our analytical methods for computing cortical networks are also well characterized and openly available (Tokariev et al., 2019a,^2021^) to allow direct benchmarking across methodologies. However, there are limitations arising from the limited numbers of study subjects available for such work, and further cohorts with prospective patient recruitments are needed to validate the present findings. The choice of individual analytical parameters is a result from an interplay between technical, biological, and physical factors. For instance, with the increase in the number of recording electrodes, it is possible to generate higher resolution cortical source matrices which, however, might be readily affected by volume conduction (Palva et al., 2018). Conversely, a low number of recording electrodes limits the source-level analysis only to the cortical areas that can be reliably reconstructed (Tokariev et al., 2016a). In either case, the analytical results need to be appropriately corrected to exclude technical factors, such as volume conduction before the results interpretation. We employed a principled simulation approach to generate estimates of reliability for each source. Moreover, using the relatively high spectral resolution in the initial phases of the analysis will include some spectral leakage across frequency bands. However, we do not expect our overall findings to be compromised by these issues because we looked for effects that span consistently across wider cortical areas and wider frequency bands. Notably, this work reports group differences and statistical correlations but it cannot provide direct evidence for a genuine causal reasoning. The work identifies human EEG correlates of SRI exposure, opening two directions of future research: First, these EEG metrics can be used as an outcome measure in larger-scale follow-up studies to define their clinical significance with respect to later neurodevelopment (Hermansen and Melinder, 2015; Man et al., 2015). Second, the observed EEG measures allow benchmarking with future preclinical studies where experimental animal models are used to disclose the underlying cellular- and molecular-level mechanisms.

## Results

First, we compared the sleep state-specific networks between the HC and SRI groups to see if there is a systematic sleep state-related effect from the SRI exposure. The overall patterns of networks appeared comparable and an edge-wise statistical comparison did not disclose any significant differences (corrected for multiple comparisons). These suggest that the potential effects of SRI exposure on the static sleep state-related networks are less than the variability in network strengths between infants.

### *In utero* Exposure to Serotonin Reuptake Inhibitor Reverts the Dynamics of Sleep Networks

Next, we wanted to see if sleep-related network dynamics, or change in network strengths between sleep states, is affected by SRI exposure. This approach was motivated by our recent works where comparable sleep-related network dynamics was shown to disclose significant effects from prematurity (Tokariev et al., 2019a) and *in utero* exposure to maternal antiepileptic medication (Tokariev et al., 2021). Such analysis of relative individual level change is more powerful in detecting salient effects because it automatically calibrates to the individual baseline levels of network strengths. Comparison of the connectivity estimates between sleep states showed that the individual level direction of change in network strengths during transition from AS to QS was different for the infants in the SRI and the control groups. We found three frequency-specific patterns showing significant sleep-by-group interaction (Figure 2A). The first two networks were observed at the neighboring theta-range frequencies Fc = 3.1 Hz (pFWE = 0.012, Cohen’s d = 0.32) and Fc = 3.7 Hz (pFWE < 0.001, Cohen’s d = 0.32). Topologically, both networks were similar and predominantly comprised interconnected clusters of areas in the frontal lobe making long-range projections to occipital cortices. The third network was in the middle of the alpha frequency range (Fc = 11.1 Hz; pFWE = 0.016, Cohen’s d = 0.35) and it was spatially more constrained, including longitudinal connections near midline in both hemispheres. These network dynamics had different direction for the lower and higher frequencies (Figure 2B). Comparison to later neurodevelopment (the Cognition score in the Bayley scales) showed that the network dynamics, or the amount of connectivity change in these patterns, was not significantly correlated to the neurodevelopmental outcome.

### Serotonin Reuptake Inhibitor Reduces Sleep-Related Discrimination in Global Phase-Amplitude Coupling at Higher Nesting Frequencies

We next computed cross-frequency distributions of global PAC for a wider range of pairwise frequency combinations (nested vs. nesting) during each sleep state (Figure 3A). The overall finding was very comparable between the groups while it differed markedly between sleep states. During AS, the highest PAC was constrained to the lowest nesting frequencies (0.5–0.6 Hz) while the nested activity spread over a wider frequency range (mostly 11–23 Hz). During QS, however, both nesting and nested frequencies (∼0.5–1.2 Hz and ∼3–33 Hz, respectively) had a much wider frequency spread compared to the findings in AS. Nevertheless, the global PAC measures did not show statistically significant group differences. Note, that the global PAC values shown on Figure 3A were computed as a mean across all cortical parcels (see Methods), nevertheless, the control analysis with medians shows similar results.

Comparison of sleep states, however, disclosed major differences across wide frequency ranges (Figure 3B). Both infant groups exhibited significantly higher PAC at QS between lower nesting frequencies (0.5–0.9 Hz) and the whole range of nested frequencies (3.1–33.1 Hz). Group wise comparison of the sleep state differences (Figure 3B) suggested a sparser PAC coupling matrix for the SRI infants, which prompted assessment with higher spatial resolution.

### Spatially Resolved Cortical Phase-Amplitude Coupling Discloses Regions Suppressed by Serotonin Reuptake Inhibitor Exposure

**FIGURE 4:**
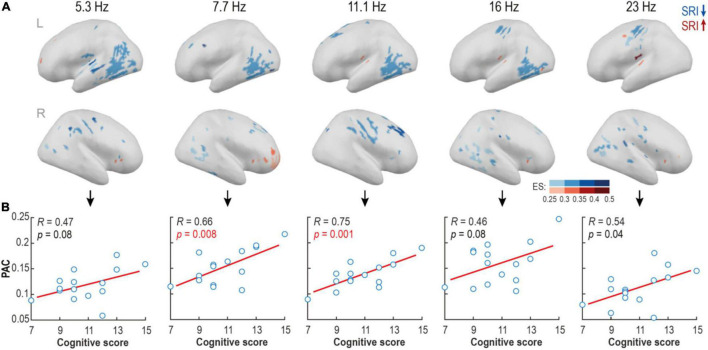
Source-level analysis reveals focal areas with suppressed phase-amplitude coupling (PAC) in serotonin reuptake inhibitor (SRI) group. **(A)** Spectrally and spatially broad differences in PAC strength were found between groups at quiet sleep. Blue colors show areas with reduced, and red areas – with elevated PAC levels in SRI cohort relative to HC. Shades code the effect size of the contrast (ES; rank-biserial correlation). **(B)** The mean PAC strength in the contrast regions positively correlated with cognitive scores at two years of age in the SRI infants. The strongest effect was observed within alpha frequency range (*p* = 0.008, pFDR = 0.02, *R* = 0.66 for Fc = 7.7; and *p* = 0.001, pFDR = 0.005, *R* = 0.75 for Fc = 11.1; Pearson test).

The spatial group differences in PAC were tested further at the level of individual cortical sources. As motivated by the above findings (Figure 3), we analyzed a broader nesting frequency band 0.5–1.2 Hz and a set of non-overlapping nested narrow bands (Fc = 5.3, 7.7, 11.1, 16, and 23 Hz). We found multiple spatially constrained cortical areas where PAC was reduced in the SRI infants. The findings were strikingly comparable across a wide range of nested frequencies (Figure 4A) suggesting a robust characteristic of the SRI effects. The most prominent reduction of PAC was seen in the posterior temporal area in the left hemisphere at nested frequencies of ∼5–16 Hz. There was also a region with reduced PAC at ∼11–23 Hz near central sulcus, as well as two regions with a reduced PAC at ∼7–16Hz at right side midfrontal and perisylvian areas. Comparison to neurocognitive outcome at 2 years showed a positive trend-level correlation in the whole frequency range (5–23 Hz; *p* ≤ 0.08 and Pearson’s *R* ≥ 0.46 for all; see Figure 4B) for the PAC estimates in all of these regions. The most significant effect was constrained to 7–11 Hz frequency range (*p* ≤ 0.008, pFDR ≤ 0.02, Pearson’s *R* ≥ 0.46 for both). Differences in PAC during AS were more scattered and they offered less obvious physiologically relevant interpretation.

## Discussion

Here we show that prenatal SRI exposure in human offspring may cause selective effects on cortical network activity that further links to neurodevelopmental outcomes. Our results are fully in line with the overall notion from the recently accumulated literature (Videman et al., 2017; Brown-Lum et al., 2020; Wisner et al., 2020; Kautzky et al., 2021) that *in utero* drug exposures may cause subtle, yet measurable changes in the brain structure and function, even if these drugs are considered safe with respect to major teratogenic sequalae. The present cortical level results extend the previous global scalp level findings (Videman et al., 2017) showing SRI effects on cross-frequency coupling in the same dataset. The lack of robust PPC changes by SRI exposure suggests that SRI affects neuronal function in the local cortical networks measured by PAC rather than the long-range cortico-cortical networks measured by PPC. More generally, our current study expands on prior literature by providing a spatially and spectrally detailed account of the SRI effects on the newborn cortical activity. In line with our previous results (Videman et al., 2017), the present study on cortical activity did not find global-level PAC differences between the groups. However, our present source-level analysis disclosed spatially more confined PAC suppression in the SRI group, which was consistent across a wider frequency band. This suggests that *in utero* pharmacological treatment may have spatially selective effects on early brain function that escape detection when analyzing at larger scale, such as cortical parcels or scalp electrodes. Here we also extend our previous findings reporting decrease in amplitudes and altered inter-hemispheric co-occurrence of bursts in SRI infants at lower frequencies (Videman et al., 2017): we show a reversed sleep-related dynamics in the phase-phase cortical networks at mid-range frequencies. The recently enabled precision of EEG analyses allows identifying specific drug effects, yielding greater effect size and better disclosing links to clinical neurodevelopmental outcomes.

The globally wide use of maternal drug treatments during pregnancy has raised many concerns about fetal neurodevelopment. These are commonly assessed by studying teratogenicity in multiple animal models and by careful evaluation of drug exposures in the human subjects (Tomson and Battino, 2012; Ponticelli and Moroni, 2018; Black et al., 2019). However, more recent clinical evidence (Ornoy, 2017; Videman et al., 2017; Wisner et al., 2020; Kautzky et al., 2021), together with the current understanding of activity-dependent brain development (Molnár et al., 2020), has prompted another wave of evaluation of fetal drug effects. It is currently more common to study less robust neurodevelopmental effects of structure and function. For instance, there may be changes in the cellular level microstructure or function in the developing animal cortex, or long term neurobehavioral effects in clinical cohorts. Recent studies have suggested multiple mechanisms for the neurodevelopmental effects of prenatal SRI exposure. Notably, the expression of serotonin transporter (5-HTT) gene is genetically scheduled, region-specific, changes during fetal development and converges toward more mature patterns only postnatally (Homberg et al., 2010; Kiryanova et al., 2013); it is also known to be affected by various hormonal factors, maternal psychiatric status or fetal drug exposure (Oberlander et al., 2009; Homberg et al., 2010; Kiryanova et al., 2013).

There is a striking lack of suitable candidate markers for translational work to support mechanistic understanding between microstructure in the animal models and neurobehavior in the human cohorts. Our choice of PAC as the measure of interest was motivated by its assumed reflection of the intracortical circuitry interactions, and its ability to generalize for probing cortico-cortical interactions in both the non-invasive human recordings and the typically invasive animal models. Prior preclinical work has shown that fetal SRI exposure (Xu et al., 2004; Homberg et al., 2010; Liao and Lee, 2011; Simpson et al., 2011) affects cortical microcircuitry and reduces cortico-cortical synchronization estimated from neural spike-timing (Simpson et al., 2011). The cross-frequency integration by PAC is assumed to rely on an effective interplay in the local cortical–subcortical (including subplate) networks (Hyafil et al., 2015). Hence a reduction in this activity is likely to reflect a deficient cortico-cortical interaction, which is compatible with the human (McGinn and Valiante, 2014) and animal (von Nicolai et al., 2014; Samiee et al., 2018) literature. The observed changes in PAC coupling by drug exposures may also be probed in the *in vitro* preparations of acute neuronal cultures (Johnson et al., 2017; Segneri et al., 2020). Intriguingly, PAC of this kind is also observed as a robust characteristic in the spontaneously developing organoids (Trujillo et al., 2019), which opens a pathway to study neurodevelopmental effects of SRIs in organoid preparations and then translate the findings back and forth to EEG recordings in live human infants.

Prior literature has generally considered that the PAC is a ubiquitous property of the early neuronal network activity (Vanhatalo et al., 2005; Li et al., 2017; Moghimi et al., 2020), while any spatial differences in the PAC mechanisms have been only studied in older infants (Mariscal et al., 2021). Our present findings could not be explained with studies on older human subjects because cortical mechanisms underlying EEG signal characteristics, including PAC, will change after neonatal period (Vanhatalo and Kaila, 2006; Simpson et al., 2011; Molnár et al., 2020; Wallois et al., 2021). Our present findings are, however, compatible with the recent experimental work showing intracortical loss of histological organization and neuronal synchrony (Xu et al., 2004; Simpson et al., 2011), both of which would conceivably link to loss of PAC. The spatial preference of the SRI effects on PAC cannot be explained by experimental literature because experimental models have routinely focused on very few cortical areas only, such as sensorimotor cortex and its thalamic connections (Xu et al., 2004; Simpson et al., 2011). Future experimental studies are needed with better cortical coverage and spatial comparisons to explain the cellular level effects seen in the human data. In addition, atlas matching of high resolution neurophysiological findings to modern neuroimaging and/or postmortem human neuroanatomy may provide intermediate steps for mechanistic explanations (Tokariev et al., 2021). They can be also interpreted using fundamental principles that appear to link different scales of neuronal activity, structure, and expression patterns (Gao et al., 2020; McColgan et al., 2021; Tokariev et al., 2021).

As to cellular level mechanisms, our present findings support the novel framework of “booster circuit activity” shown in the experimental animal literature (Murata and Colonnese, 2016; Kirischuk et al., 2017): The cortico-cortical or cortico-thalamic loops would amplify sensory signals in such booster circuits to enhance the signal-to-noise ratios that are needed for improving activity-dependent organization of neuronal networks (Petersson et al., 2003; Molnár et al., 2020). Consequently, lower PAC seen in our SRI exposed infants relative to control group would indicate a general compromise in this process; the strength of PAC in the affected cortical regions was found to link to the levels of later emerging global cognitive levels, which suggests a very long neurodevelopmental trajectory from the early network interactions to long-term global cognitive functions.

The clinical significance of our present PAC findings is supported by the observed robust correlations of PAC levels to the long-term neurodevelopmental outcomes. More broadly, our study advocates for the potential use of PAC as a clinically potent measure of cortical function that may reflect effects of past adversities to future neurodevelopment. At the same time, PAC can be directly applied to different levels of experimental models and its underlying mechanisms can be assessed at the cellular level, which makes it very suitable for bridging the inevitable gap between animal and human studies. Clinically, our study may support a critical attitude toward the use of maternal treatment with SRIs during pregnancy (Hanley et al., 2015; Kautzky et al., 2021). Recognizing this will call for re-evaluation of treatment guidelines, including the optimal balance between pharmacological and psychotherapeutic approaches (Cosci et al., 2020a,b).

## Data Availability Statement

The datasets presented in this article are not openly available, but the clinical EEG data and neurocognitive performance assessments can be made available via data sharing agreement with Helsinki University Hospital. Connectivity matrices for all subjects are available by request to the corresponding author AT, anton.tokariev@helsinki.fi.

## Ethics Statement

The studies involving human participants were reviewed and approved by the Ethics Committee of the Helsinki University Hospital. Written informed consent to participate in this study was provided by the participants’ legal guardian or next of kin.

## Author Contributions

AT, MV, and SV contributed to conception and design of the study. MV contributed to data collection. VO organized dataset and pre-processed the data. AT developed and implemented analytical tools, analyzed data, and prepared figures. AT and SV wrote the first draft of the manuscript. All authors contributed to manuscript revision, read, and approved the submitted version.

## Conflict of Interest

The authors declare that the research was conducted in the absence of any commercial or financial relationships that could be construed as a potential conflict of interest.

## Publisher’s Note

All claims expressed in this article are solely those of the authors and do not necessarily represent those of their affiliated organizations, or those of the publisher, the editors and the reviewers. Any product that may be evaluated in this article, or claim that may be made by its manufacturer, is not guaranteed or endorsed by the publisher.

## Footnotes

1
https://www.ncbi.nlm.nih.gov/pmc/articles/PMC7940391/
